# The Spectrum of Postoperative Complications and Outcomes After Pancreaticoduodenectomy: A Retrospective Outlook From a Developing Country

**DOI:** 10.7759/cureus.22218

**Published:** 2022-02-14

**Authors:** Abdullah Bin Zubair, Ismail Abdur Rahman Khan Sherwani, Muhammad Ahmad, Mohammed Ahmad Tahir, Muhammad Ibrahim Khalil, Mohammad Mudassar Bukhari, Muzammil Sabir, Assadullah A Bhatti, Nitasha Afzal, Mehwish Kaneez

**Affiliations:** 1 Surgery, Rashid Latif Medical College, Lahore, PAK; 2 Surgery, Bahawal Victoria Hospital, Bahawalpur, PAK; 3 Surgery, University College of Medicine and Dentistry, Lahore, PAK; 4 Surgery, Rawalpindi Medical University, Rawalpindi, PAK

**Keywords:** periampullary tumors, postoperative complications, postoperative outcomes, pancreatic tumors, obstructive jaundice, pancreaticoduodenectomy

## Abstract

Background

Pancreaticoduodenectomy is an extremely complex surgical procedure that mandates aggressive postoperative management. Unfortunately, in developing countries, the limited resources and poor postoperative care lead to multiple complications and abysmal outcomes. Therefore, our study aimed to evaluate the spectrum of postoperative complications and outcomes among patients undergoing pancreaticoduodenectomy.

Methods

This retrospective study involved a total of 97 patients who underwent pancreaticoduodenectomy for ampullary, periampullary, or pancreatic tumors. Patients with advanced metastasis and unresectable tumors were excluded from the study. Patients were studied for various parameters including the demographic details, postoperative outcomes, characteristics of the tumor, and postoperative complications.

Results

Out of 97 patients, 59 (60.8%) patients were males. The mean age of the study participants was 53.43 ± 17.89 years. Jaundice and abdominal pain were the most common presenting symptoms among the study participants. Of the 97 patients, 58 (59.8%) had malignant tumors. A total of 49 patients developed various postoperative complications including surgical site infections (10.3%), anastomosis leakage (9.27%), pancreatic fistula (9.27%), cholangitis (7.2%), and biliary leakage (4.1%). A total of 29 (29.9%) patients expired due to postoperative complications.

Conclusions

Surgical site infections, anastomosis leakage, pancreatic fistula, cholangitis, and biliary leakage are common but preventable postoperative complications after pancreaticoduodenectomy. These lead to morbidity and mortality, especially in the setting of a resource-deprived developing country. Aggressive postoperative management, improved surgical technique, better intraoperative hemostasis management, and a multi-disciplinary approach for the management of such patients can help in preventing postoperative complications and improving the postoperative outcomes.

## Introduction

Pancreaticoduodenectomy is considered one of the most complex surgical procedures in hepatobiliary surgeries due to major organ resections and complicated gut anastomosis [1]. It is commonly indicated for a myriad of biliary, ampullary, pancreatic, and periampullary neoplasms [2]. Periampullary carcinomas have a dismal prognosis and are ranked as the fourth leading cause of cancer-related mortalities worldwide [3]. The risk factors associated with the incidence of pancreatic head cancers, ampullary and periampullary carcinomas increased due to age, ethnicity, use of tobacco, alcoholism, chronic pancreatitis family history, familial adenomatous polyposis syndrome (FAP), and genetic susceptibility [2].

Most of the patients present at an advanced stage of the tumor with only 10-20% of patients presenting with a tumor that can be resected [4]. Despite the recent advances in adjuvant therapy, the best and concrete way to improve survival in patients with periampullary cancers is comprehensive oncological resection [5]. Thus, pancreaticoduodenectomy is considered as a curative surgery for ampullary, periampullary, and pancreatic head cancers which includes complete gross and microscopic resection of tumor elements [6,7]. It is a very complicated procedure with relatively high morbidity and mortality rates even in highly developed hospitals. The morbidity rate ranges from 30% to 60% while the mortality rate has been recently decreased from 25% to less than 5% [6]. Despite a significant decline in mortality rate, the five-year survival rate after the procedure is still up to 10-20% [7].

Moreover, pancreaticoduodenectomy is associated with a large number of postoperative complications that influence survival. These complications include hemorrhage, pancreatic fistula, bile leak, intra-abdominal abscess, delayed gastric emptying, and surgical site infection [5]. These complications can be life-threatening, may require multiple interventions, delay recovery, increase hospital stay and also have an impact on the psychological state [8]. The incidence of postoperative complications has considerably decreased in developed countries due to advancements in perioperative surgical techniques and improved postoperative care. However, developing countries still struggle to provide optimal care to such patients due to limited resources [8,9]. Moreover, developing countries account for a major proportion of the global burden of poor surgical outcomes [9].

These complications following pancreaticoduodenectomy significantly alter the clinical course of patients. Additionally, the lack of research and poor facilities in developing countries demand the need for this study so that the exact prevalence of postoperative complications in such patients can be assessed. This study aimed to evaluate the spectrum of postoperative complications and postoperative outcomes which will help to reduce the procedure-related mortality following pancreaticoduodenectomy so that clinical outcomes after surgery can be improved.

## Materials and methods

This retrospective study was conducted at the Department of General Surgery, District Headquarter Hospital, Rawalpindi, Pakistan. The study included data from all patients that underwent pancreaticoduodenectomy from August 2015 till December 2021. All patients having various presenting symptoms including obstructive jaundice, unexplained weight loss, severe vomiting, and computed tomography (CT) scan proven mass in the ampullary, periampullary, or pancreatic head region were included in the study. Patients having complete encasement of the superior mesenteric artery, portal vein, and celiac artery were excluded from the study. Moreover, patients with severe multi-visceral involvement and distant metastasis were considered surgically unresectable tumors. Therefore, such patients were also subjected to exclusion. Finally, a total of 97 patients were part of the final data analysis. The ethical approval was obtained from the institutional research board of Rawalpindi Medical University, Rawalpindi, Pakistan with the approval number IRB-2022-SUR-022.

Demographic details of the study participants including gender, age, presenting symptoms, and comorbidities were reported. Patients were studied for various parameters including the tumor location, grading, and histopathological subtype of the tumor. The outcome of pancreaticoduodenectomy one month after the surgery and the spectrum of postoperative complications were also studied. Thereafter, the data was entered and analyzed using Statistical Package for Social Sciences (SPSS) version 25 (Armonk, NY: IBM Corp.).

## Results

In the present study analyzing 97 patients, the mean age was 53.43 ± 17.89 years with a range of 31-82 years. Most of the patients had multiple presenting symptoms and comorbidities. The demographics of the study participants are delineated in Table 1.

**Table 1:** Breakdown of characteristics of study participants.

Parameters		Frequency	Percentages
Gender	Male	59	60.8%
	Female	38	39.2%
Age groups	30-40 years	7	7.2%
	41-50 years	18	18.5%
	51-60 years	37	38.1%
	60-70 years	19	19.6%
	More than 70 years	16	16.5%
Presenting symptoms	Jaundice	71	73.1%
	Vomiting	55	56.7%
	Abdominal pain	62	63.9%
	Unexplained weight loss	45	46.4%
	Fever	31	31.9%
	Palpable abdominal mass	23	23.7%
Comorbidities	Diabetes mellitus	47	48.4%
	Hypertension	39	40.2%
	Ischemic heart disease	19	19.5%
	Asthma	4	4.1%

Initial diagnosis of the patients was based upon baseline, clinical, and radiological investigations. Further highlights regarding the tumor site, grade, and other histopathological parameters are shown in Table 2.

**Table 2:** Elucidation of histopathological details of the resected tumors.

Parameter		Frequency	Percentages
Tumor site	Ampullary	31	32%
	Periampullary	27	27.8%
	Pancreatic	19	19.6%
	Duodenal	15	15.5%
	Extrahepatic biliary tree	5	5.1%
Tumor grade	Well-differentiated	8	8.2%
	Moderately differentiated	62	63.9%
	Poorly differentiated	25	25.7%
	Undifferentiated/anaplastic	2	2%
Involvement of lymph nodes	Yes	49	50.5%
	No	48	49.4%
Perineural invasion	Yes	41	42.2%
	No	56	57.7%
Nature of tumor	Benign	39	40.2%
	Malignant	58	59.8%

The mean length of hospital stay of the patients was 28.71 ± 5.3 days. Out of 97 patients, 29 succumbed to postoperative complications (in-hospital mortality). On six months follow-up, 12 more patients died due to advanced metastasis and disease complications. The spectrum of postoperative outcomes after the surgery is elucidated in Table 3.

**Table 3:** Delineation of postoperative outcomes among the study participants.

Postoperative outcomes		Frequency	Percentages
Need for blood transfusion	Yes	88	90.7%
	No	9	9.2%
Need for re-exploration	Yes	13	13.4%
	No	84	86.6%
In-hospital mortality	Yes	29	29.9%
	No	68	70.1%
Need for adjuvant chemotherapy	Yes	25	25.8%
	No	72	74.2%
Need for mechanical ventilation	Yes	37	38.1%
	No	60	61.9%
Need for adjuvant radiotherapy	Yes	25	25.8%
	No	72	74.2%

Out of 97 patients, 49 (50.5%) developed immediate several major postoperative complications. Postoperative cholangitis, surgical site infections, anastomosis leakage, and pancreatic fistula were some of the few complications that were reported among the study participants. The breakdown of various postoperative complications after pancreaticoduodenectomy is shown in Table 4.

**Table 4:** Spectrum of postoperative complications among the patients.

Postoperative complications	Frequency	Percentages
Surgical site infections	10	10.3%
Acute respiratory distress	3	3.1%
Pancreatic fistula	9	9.3%
Anastomosis leakage	9	9.3%
Incisional hernia	1	1.0%
Delayed gastric emptying	6	6.2%
Biliary leakage	4	4.1%
Cholangitis	7	7.2%
Total	49	50.5%

## Discussion

Pancreaticoduodenectomy is a complex procedure that involves resection of the gallbladder, bile duct, first part of the duodenum, and head of the pancreas [1-5]. It demands a meticulous surgical technique due to complex anastomosis (hepaticojejunostomy, gastrojejunostomy, and pancreaticojejunostomy). This procedure is associated with high postoperative morbidity and mortality, especially in developing countries, and therefore demands invasive postoperative management [3]. Nonetheless, the procedure remains the treatment of choice for curative management of non-complicated and non-metastatic ampullary or periampullary neoplasms [7,10].

In our study, 60.8% of the patients who underwent pancreaticoduodenectomy were elderly males. These demographics are in comparison to another study done in Pakistan which shows a male predominance of 66.6% [3]. Another study also validates this finding and shows that periampullary and pancreatic cancers have a male preponderance [10]. The most common presenting symptoms were jaundice, abdominal pain, vomiting, and weight loss. Another study done in Iraq showed similar results where 82.6% of the patients had jaundice while 77.6% had weight loss and anorexia as presenting symptoms [5]. Our study revealed that the majority of the patients undergoing surgery were aged between 51 and 60 years. Other studies from developing countries also elucidated similar results where nearly 30% of the patients were aged between 51 and 60 years [3-5].

The evaluation of histopathology of the resected specimen in our study showed that ampullary carcinoma was the most frequently diagnosed tumor, followed by periampullary, pancreatic, and duodenal cancer. A study from India showed that 67.7% of the specimen upon Whipple's resection were ampullary and periampullary lesions [11]. On the contrary, a study done in Australia reported that most patients who underwent pancreaticoduodenectomy suffered from carcinoma of the pancreatic head [12]. The difference might be due to differences in environment, genetics, race, and setting of a developed country.

Ampullary and periampullary cancers show early signs of persistent obstructive jaundice due to close anatomical vicinity with the extrahepatic biliary channels [11,12]. Small-sized ampullary cancer leads to an early biliary obstruction that causes symptoms such as nausea, vomiting, abdominal pain, and jaundice [13]. On the other hand, pancreatic and duodenal cancers have to reach a certain size before signs and symptoms of biliary obstruction occur [13,14]. Furthermore, upon evaluation of the grade of the resected specimen, it was concluded that most of the patients were suffering from poorly differentiated tumors (suggestive of malignancy). Another study from Pakistan showed the predominance of malignant periampullary and ampullary carcinomas with nodal and perineural invasion [1].

One of the critical outcomes of pancreaticoduodenectomy in our study was the need for re-exploration. It was needed in a significant number of patients because of several complications such as biliary leakage, anastomosis leakage, and pancreatic fistula. Nearly 9.3% of the patients developed anastomosis leakage and pancreatic fistula while 4.1% of the patients developed biliary leakage. Another study showed that nearly 18% of the patients developed anastomosis leakage following pancreaticoduodenectomy [14]. The difference might be due to different clinical setups and surgical approaches. Nevertheless, it cannot be ignored that re-exploration predisposes the patients to additional surgical trauma, complications, morbidity, and mortality.

Another common but preventable complication that we observed was the development of surgical site infections in 10.3% of the patients. A study from Pakistan illustrated that 7% of the patients developed surgical site infection, a finding approximately close to ours [3]. However, another study reported that 32% of the patients developed surgical site infections following pancreaticoduodenectomy [15]. Pancreatic fistula and the presence of preoperative cholangitis are reported to be important predictors of organ space surgical site infection following pancreaticoduodenectomy [3,15].

In our study, 29.9% of the patients succumbed after the procedure. A study carried out from Japan showed a markedly lower mortality rate of 3.3%. [16]. The lower mortality rate following pancreaticoduodenectomy in developed countries shows that meticulous surgical techniques and appropriate postoperative management can aid in yielding favorable outcomes. Nonetheless, an increased patient load, inadequate resources, and insufficient critical care management tools affect the postoperative outcomes. Even with these challenges, surgeons in developing countries can avoid these complications with improvement in surgical techniques, aggressive postoperative monitoring, and combined effort through a multidisciplinary approach.

Even with the complexity of the procedure, the above-mentioned postoperative complications can be prevented. Appropriate preoperative optimization of the patients and administration of broad-spectrum antibiotics postoperatively can help reduce the chances of bacterial cholangitis and prevent organ space surgical site infections [7,10,15]. Moreover, incisional surgical site infections can be prevented with better postoperative care, daily dressings, improved wound management, and attempts to reduce pancreatic fistula formation. Improvement of surgical techniques and intraoperative hemostasis can help to prevent anastomosis leakage and reduce the need for re-exploration. 

Even though our study highlights important findings it has certain limitations. It was a single-centered study with a retrospective study design so associations of complications with the cause could not be developed. The multi-centered prospective cohort studies on the topic will aid in a better understanding of postoperative complications following pancreaticoduodenectomy. Moreover, we used a convenience sampling technique for the selection of study participants which accounts for another limitation. Nonetheless, our study yields important conclusions, especially for the development of hepatobiliary surgeries in developing countries.

## Conclusions

Anastomosis leakage, pancreatic fistula, and surgical site infections are the most common complications observed in patients undergoing pancreaticoduodenectomy. The complications and mortality after the procedure have dramatically decreased in the western world; however, in resource-deprived nations such as Pakistan, the mortality rate due to postoperative complications is alarmingly high. Appropriate postoperative management, improved surgical technique, better intraoperative hemostasis management, and a multi-disciplinary approach for the management of such patients can help in improving the postoperative outcomes.
